# A Study to Assess the Impact of Education on the Knowledge and Attitude Toward Cervical Cancer and HPV (Human Papillomavirus) Vaccination Among Female Healthcare Students

**DOI:** 10.7759/cureus.59856

**Published:** 2024-05-08

**Authors:** Ishan Verma, Rashmi Bajpai, Vibha Arjaria, Lekhraj Garg, Ayushi Mungad, Devendra Singh, Jai Gavli, Apurva Khare

**Affiliations:** 1 General Medicine, All India Institute of Medical Sciences, Nagpur, Nagpur, IND; 2 General Medicine, LN Medical College and Research Center, Bhopal, IND; 3 Obstetrics and Gynaecology, LN Medical College and Research Center, Bhopal, IND; 4 Epidemiology and Public Health, LN Medical College and Research Center, Bhopal, IND; 5 General Medicine, LN Medical College and Research Center, BHOPAL, IND; 6 Cardiology, Saveetha Medical College and Hospital, Chennai, IND; 7 General Medicine/Rheumatology, LN Medical College and Research Center, Bhopal, IND

**Keywords:** attitude, knowledge, female, students, professionals, health care, cancer, hpv vaccine, cervical cancer

## Abstract

Background

Cervical cancer ranks among the top five cancers in India, with human papillomavirus (HPV) types 16 and 18 causing up to 70% of related lesions. HPV infection, acquired through various routes, poses risks for both men and women, especially in the age group of 16 to 25 years. Effective prevention is possible through HPV vaccination, with Cervarix and Gardasil approved for use in India. Despite its proven efficacy, HPV vaccine use remains minimal. This study aims to evaluate awareness, willingness, and barriers among female healthcare students while assessing the impact of a health education program on their knowledge and attitude.

Methods

The present study is an educational interventional study conducted on 489 female students in the healthcare sector in the age group of 19-25 years. Two questionnaires (pre-lecture and post-lecture) were used. After the pre-lecture questionnaire, a session on cervical cancer and vaccine education was delivered by the subject expert. Afterward, the post-lecture questionnaire was given and the impact of session was analyzed using various statistical tools.

Result

A total of 489 students across MBBS (Bachelor of Medicine, Bachelor of Surgery), BAMS (Bachelor of Ayurvedic Medicine and Surgery), BHMS (Bachelor of Homoeopathic Medicine and Surgery), paramedical, and nursing courses participated in the study. Prior to the lecture, knowledge regarding cervical cancer and vaccines was generally low across subgroups, witnessing improvements ranging from 60% to 100% in various questionnaire sections post-education. Understanding of the importance of a Pap smear (Papanicolaou test) increased significantly from 21% to 79% after the educational session. The most preferred measure to boost coverage was the inclusion of the HPV vaccine in the National Immunization Schedule, with lack of awareness identified as the most significant barrier.

Conclusion

An educational session not only enhances knowledge but also boosts willingness for cervical cancer vaccination. Inclusion of the vaccine in the National Immunization Schedule not only increases acceptability but also indirectly raises awareness.

## Introduction

Cervical cancer is among the top five leading cancers in India and is the second most common cancer in females in India. Cervical cancer accounted for around 9% deaths of all deaths in India, thus becoming the second most common cause of death from cancer in India [1]. Worldwide data show that the incidence of cervical cancer ranks 8th among all cancers and ranks fourth among all cancers on females [2,3]. Almost all cases of cervical cancer are caused by human papillomavirus (HPV). HPV group of viruses consists of more than 100 types of viruses, of which at least 14 types are high-risk and are known to cause various cancers including cervix, vulva, anus, vagina, and oropharynx. HPV types 16 and 18 cause up to 70% of cancerous and precancerous lesions in the cervix [2]. HPV infection can be acquired in both men and women through various sexual (most common route) and non-sexual routes [4]. Probability of acquiring infection and of developing cervical cancer increases further with factors such as poor personal hygiene, multiple pregnancies, multiple sexual partners, early age of marriage, and smoking [5]. The age group of 16 to 25 years is most vulnerable. Around 80% of the infections resolve spontaneously. Out of the remaining 20%, most infections result in benign lesions, while only less than 5% of the infections result in cervical cancer [6]. Cervical cancer can largely be prevented through HPV vaccination. The protective efficacy of HPV vaccination (almost 90-99%) is well established [7]. HPV vaccination has been available since 2006-2007 and was first introduced in India in 2008-2009 [8,9]. Two types of HPV vaccine are available, Cervarix (Bivalent vaccine) and Gardasil (Quadrivalent vaccine),- and both are licensed for use in India [10]. Despite the proven role of HPV vaccination in the prevention of cervical cancer, use of HPV vaccination as a preventive tool is minimal in India. HPV vaccination, though recommended by various authorities including the World Health Organization (WHO) and the Indian Academy of Pediatrics, is not a part of the National Immunization Schedule (NIS) of India at the time of data collection [11-13]. The awareness and acceptance of HPV vaccine in the general population is limited and largely influenced by cost, sociocultural factors, and paucity of awareness. Several studies conducted in various states of India among different population categories (including medical students) have shown poor awareness about cervical cancer and HPV vaccination [7,8,14,15].

By the time a girl attains sexual maturity, she should be well aware of the female genital tract and its common diseases, as well as the mode of its prevention. This expectation from female students in the health sector becomes even higher as compared to the girls from the non-medical sector. In the Indian context, individuals involved in healthcare, including doctors, nurses, AYUSH (Ayurveda, Yoga and Naturopathy, Unani, Siddha, and Homeopathy) practitioners, and laboratory technicians, often become trusted health advisors within their communities. This responsibility is particularly crucial regarding topics such as cervical cancer and HPV vaccination, given the significant impact these diseases can have. Considering that the optimal age for vaccination is 12 years, it is vital to ensure that correct information and motivation for vaccination are conveyed early to both eligible individuals and their parents. This includes educating school teachers, who play a pivotal role in disseminating health-related information. Social media and government programs are expected to add more awareness in this regard. Focusing on females aged 19 to 25 years in our study was deliberate. This age group encompasses healthcare professionals who are expected to advocate for vaccination. By ensuring their own vaccination, they are better positioned to convey accurate information and motivate others in their communities to seek vaccination.

With the present study, we aim to assess the current awareness, willingness, and barriers toward HPV vaccine among female students in the healthcare sector. Simultaneously, this study also assesses the impact of health education program on their knowledge and attitude.

## Materials and methods

The present study is an educational interventional study that was conducted from February to March 2021 in various medical and nursing colleges situated in a city of Madhya Pradesh, India. The participants were female students in the healthcare sector in age the group of 19 to 25 years. Those who did not provide consent or had a current or past history of cervical cancer were not included in the study. The study received approval from the Institutional Ethics Committee. An informed, written consent was obtained from each participant. A sample size of more than 400 was calculated based on 50% as prevalence of unknown (percentage of young females knowing about the cervical cancer vaccine is not available) at 95% confidence interval and 5% error. Convenience sampling was employed for data collection, with prior permission obtained from the respective college administration. Participants were not informed about the date, time, and agenda of the questionnaire in advance.

Two questionnaires (pre-lecture (Appendix A) and post-lecture (Appendix B) were created for the study and validated by English language experts and clinical professionals. The questionnaires were developed by reviewing previous literature, guidelines from the WHO, and guidelines of the Ministry of Health And Family Welfare, India, in consultation with experts from different fields to check the relevance and make necessary changes according to the study requirements. Thirty questions assessed knowledge, attitude, and practices regarding HPV vaccine and cervical cancer. Expert and subject reviews, using a Likert scale, guided question selection. Cohen’s Kappa Index determined 25 questions with substantial agreement for final inclusion.

Peer review by subject experts ensured questionnaire validation. Thirty females (19 to 25 years of age) underwent pilot testing, leading to necessary adjustments for further validation. The first questionnaire collected sociodemographic data and included 21 questions on cervical cancer and HPV awareness. The second questionnaire comprised 25 questions, including 18 from the first questionnaire and seven new ones. After ample time for completion, a 30-minute interactive health talk on cervical cancer and prevention was conducted by a subject expert. Subsequently, a second questionnaire gauged participants' willingness to take the vaccine and their ideas on overcoming barriers to widespread HPV vaccine implementation in India. Responses were in "yes/no," "multiple choice," or arranged in descending order, as per question requirements.

Data collected through questionnaires were tabulated and analyzed using various statistical methods. Statistical analysis was carried out using the Statistical Package for Social Sciences (SPSS) Version 21.0 (IBM Corp., Armonk, NY) and Microsoft Excel (Microsoft, Redmond, WA).

## Results

**Figure 1:**
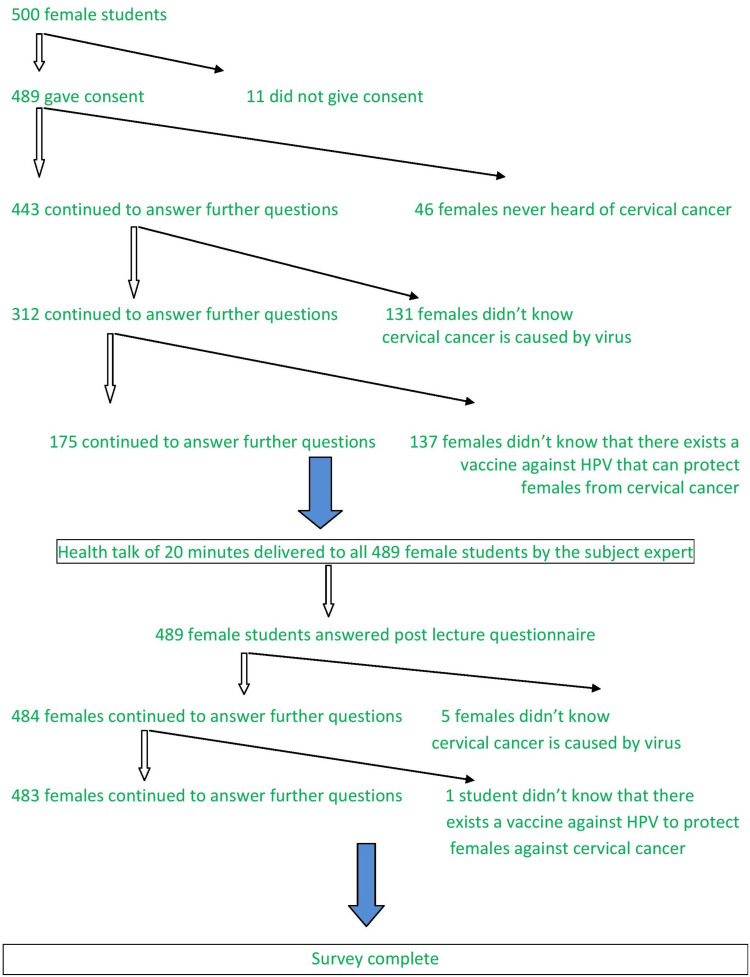
Study algorithm

Figure 1 depicts the algorithm of data collection.

In the demographic profile of the participants (Table 1), the average age was 21 years, with the majority being unmarried. The distribution among various medical courses such as MBBS (Bachelor of Medicine and Bachelor of Surgery), BAMS (Bachelor of Ayurvedic Medicine and Surgery), BHMS (Bachelor of Homoeopathic Medicine and Surgery), paramedical, and nursing was nearly equal. Notably, only 4% of participants' mothers were found to be illiterate. Furthermore, a substantial 90% of participants reported no family history of any type of cancer. This insightful information provides a comprehensive overview of the diverse and well-represented participant demographics in the study.

**Table 1:** Sociodemographic characteristics of the participants MBBS, Bachelor of Medicine, Bachelor of Surgery; BAMS, Bachelor of Ayurvedic Medicine and Surgery; BHMS, Bachelor of Homoeopathic Medicine and Surgery; INR, Indian Rupee

Parameter	Frequency (percentage), n=489
Mean age (in years)	21.2 years
Marital status	
Married	6 (1.2%)
Unmarried	483 (98.7%)
Educational course pursuing	
MBBS	96 (19.6%)
BAMS	135 (27.6%)
BHMS	65 (13.3%)
Paramedical (laboratory technician)	126 (25.7%)
Nursing	67 (13.7%)
Mother’s education	
Illiterate	21 (4.3%)
Primary school	63 (12.9%)
Secondary school	103 (21%)
12th pass	4 (0.8%)
Graduation	192 (39.3%)
Post-graduation	106 (21.7%)
Father’s education	
Illiterate	6 (1.2%)
Primary school	28 (5.7%)
Secondary school	68 (14%)
12th pass	2 (0.4%)
Graduation	203 (41.5%)
Post-graduation	182 (37.2%)
Family income (monthly income in INR)	
Below INR 20,000	63 (12.9%)
INR 20,000 to 50,000	171 (35%)
INR 50,000 to 100,000	118 (24.1%)
Above INR 100,000	137 (28%)
Family history of any type of cancer	
Yes	51 (10.4%)
No	438 (89.5%)
If yes, which cancer?	
Breast cancer	14
Ovarian/cervical/uterine cancer	2
Others	35

Completion rates for the pre-lecture questionnaire were consistently below 50% across medical student categories. Awareness of cervical cancer was widespread, except among 27% of participants in the paramedical fraternity. In the BAMS category, 35% were unaware of the causative organism, while 32% of MBBS participants did not know about the unavailability of a cervical cancer vaccine. Strikingly, no BAMS participants were vaccinated, and in other categories, less than 5% received the cervical cancer vaccine, revealing crucial areas for targeted education and intervention (Table 2).

**Table 2:** Participation and knowledge about cervical cancer in the pre-lecture questionnaire (including vaccination status of participants) MBBS, Bachelor of Medicine, Bachelor of Surgery; BAMS, Bachelor of Ayurvedic Medicine and Surgery; BHMS, Bachelor of Homoeopathic Medicine and Surgery; HPV, human papillomavirus

Questions	Overall, n=489	MBBS, n=96	BAMS, n= 135	BHMS n= 65	Paramedical, n= 126	Nursing, n=67
Participants completed the pre-lecture questionnaire	174 (35%)	35 (36%)	42 (31%)	38 (48%)	36 (29%)	23 (34%)
Never heard of cervical cancer	46 (9%)	3 (3%)	6 (4%)	0 (0%)	34 (27%)	3 (4%)
Not aware of the causative organism	131 (27%)	26 (27%)	47(35%)	3 (2%)	33 (26%)	22 (33%)
Not aware of the existence of vaccine for cervical cancer	137 (28%)	31 (32%)	40 (30%)	24 (18%)	23 (18%)	19 (28%)
Are you vaccinated with HPV vaccine?	7 (1.4%)	4 (4%)	0 (0%)	1 (0.7%)	1 (0.8%)	1 (1.5%)

When participants were asked about their preferred sources of knowledge on cervical cancer and its vaccine, books and websites emerged as the top choices across all groups. Notably, participants were allowed to select multiple options (Table 3).

**Table 3:** Sources of knowledge for cervical cancer and vaccination HCW, healthcare worker; MBBS, Bachelor of Medicine, Bachelor of Surgery; BAMS, Bachelor of Ayurvedic Medicine and Surgery; BHMS, Bachelor of Homoeopathic Medicine and Surgery

Total respondents	Social media	Books and websites	friends and family	Doctors and HCW	Newspaper
MBBS (n=96)	39	73	31	32	17
BAMS (n=135)	82	93	21	41	26
BHMS (n=65)	29	59	9	38	4
Paramedical (n=126)	45	62	16	21	12
Nursing (n=67)	8	48	4	21	4

Pre-lecture and post-lecture questions were nearly identical, focusing on cervical cancer and its vaccination. Notable shifts in knowledge were observed. Initially, 71% of paramedical participants correctly identified the affected organ, rising to 100% post-lecture. Regarding the prevalence of cervical cancer, MBBS pre-lecture scores were highest, while post-lecture, more than 60% of participants across groups answered correctly.

Out of total participants who had heard of cervical cancer and proceeded to answer pre-lecture questionnaire, only 15% of them could correctly mark all the risk factors for cervical cancer. Of these maximum correct respondents were from the BHMS group (31%) as compared to 7% from the MBBS group. Post-lecture, MBBS participants showed the highest correct responses, while nursing students remained at 19%. When asked about symptoms of cervical cancer, pre-lecture, BAMS students answered in maximum numbers (53%), but the overall correct respondents were only 21%. Post-lecture, the overall correct responses increased to 67% with maximum responses from the MBBS group (81%), while only 40% nursing students could answer correctly.

Pre-lecture, overall, 56% participants correctly knew the virus that causes cervical cancer. Out of these, maximum correct responses were from the BHMS group (95%). Post-lecture, all groups were able to answer the causative agent for cervical cancer correctly, with an accuracy reaching 95-100%. Understanding peak HPV infection age saw post-lecture improvements, notably in the MBBS, BAMS, and BHMS groups.

The ideal age for HPV vaccination was initially misunderstood by 90%, drastically improving post-lecture. Dose regimen awareness increased post-lecture, with MBBS, BAMS, and BHMS exceeding 60%, while paramedical and nursing reached 51% and 36%, respectively (Table 4).

**Table 4:** Impact of educational intervention on cervical cancer knowledge (pre- and post-lecture responses) MBBS, Bachelor of Medicine, Bachelor of Surgery; BAMS, Bachelor of Ayurvedic Medicine and Surgery; BHMS, Bachelor of Homoeopathic Medicine and Surgery; HPV, human papillomavirus; NIS, National immunization schedule; GOI, Government of India

Questions	Correct responses	Overall	MBBS, n=96	BAMS, n=135	BHMS, n=65	Paramedical, n=126	Nursing, n=67
Cervical cancer affects which organ?	Pre-lecture	434 (89%)	93 (97%)	123 (91%)	65 (100%)	90 (71%)	63 (94%)
	Post-lecture	488 (99.7%)	96 (100%)	134 (99%)	65 (100%)	126 (100%)	67 (100%)
Is cervical cancer common in developed countries?	Pre-lecture	186 (38%)	58 (60%)	47 (35%)	22 (34%)	41 (33%)	18 (27%)
	Post-lecture	348 (71%)	87 (91%)	85 (63%)	48 (74%)	77 (61%)	51 (76%)
What are the risk factors for cervical cancer? (fully correct)	Pre-lecture	75 (15%)	7 (7%)	29 (30%)	20 (31%)	13 (10%)	6 (9%)
	Post-lecture	234 (48%)	70 (73%)	59 (48%)	41 (63%)	51 (40%)	13 (19%)
What are the cervical cancer symptoms? (fully correct)	Pre-lecture	104 (21%)	2 (2%)	71 (53%)	6 (9%)	18 (14%)	7 (10%)
	Post-lecture	316 (67%)	78 (81%)	80 (59%)	51 (78%)	80 (63%)	27 (40%)
Which virus causes cervical cancer?	Pre-lecture	276 (56%)	67 (70%)	59 (44%)	62 (95%)	54 (43%)	34 (51%)
	Post-lecture	474 (97%)	96 (100%)	130 (96%)	65 (100%)	122 (97%)	61 (91%)
What is the route of transmission for HPV?	Pre-lecture	266 (54%)	65 (68%)	61 (45%)	60 (92%)	49 (39%)	31 (46%)
	Post-lecture	483 (99%)	96 (100%)	134 (99%)	65 (100%)	122 (97%)	66 (99%)
What is the peak age for acquiring HPV?	Pre-lecture	172 (35%)	44 (46%)	47 (35%)	38 (58%)	22 (17%)	21 (31%)
	Post-lecture	385 (79%)	83 (86%)	111 (82%)	53 (82%)	95 (75%)	43 (64%)
What is the time period of progression to cervical cancer?	Pre-lecture	56 (11%)	7 (7%)	21 (16%)	11 (17%)	4 (3%)	13 (19%)
	Post-lecture	353 (72%)	74 (74%)	91 (67%)	48 (74%)	97 (77%)	43 (64%)
Ideal age for HPV vaccination?	Pre-lecture	32 (7%)	7 (7%)	7 (5%)	9 (14%)	6 (5%)	3 (4%)
	Post-lecture	422 (86%)	88 (92%)	106 (79%)	62 (95%)	119 (94%)	47 (70%)
How many types of HPV vaccines are available?	Pre-lecture	85 (17%)	21 (22%)	18 (13%)	17 (26%)	18 (14%)	11 (16%)
	Post-lecture	403 (82%)	85 (89%)	113 (84%)	56 (86%)	107 (85%)	42 (63%)
What is the national NIS-GOI?	Pre-lecture	65 (13%)	7 (7%)	10 (7%)	25 (38%)	19 (15%)	4 (6%)
	Post-lecture	348 (71%)	64 (67%)	87 (64%)	57 (88%)	95 (75%)	45 (67%)
How many doses of HPV vaccine are required?	Pre-lecture	42 (9%)	9 (9%)	7 (5%)	13 (20%)	8 (6%)	5 (7%)
	Post-lecture	288 (59%)	65 (68%)	96 (71%)	39 (60%)	64 (51%)	24 (36%)

Prior to the lecture, merely 21% of students acknowledged the necessity of a Pap smear (Papanicolaou test) for sexually active women. After the session, this awareness significantly increased, with 79% of students expressing agreement with this fact.

Regarding cost awareness pre-lecture, only 5% of students were informed. Post-lecture, there was an improvement, but still, only 40% of students accurately responded to the cost-related question (Table 5).

**Table 5:** Pre- and post-lecture attitude toward cervical cancer and vaccination MBBS, Bachelor of Medicine, Bachelor of Surgery; BAMS, Bachelor of Ayurvedic Medicine and Surgery; BHMS, Bachelor of Homoeopathic Medicine and Surgery; HPV, human papillomavirus; Pap smear, Papanicolaou test; HPV, human papillomavirus

Questions	Correct responses	Overall, n=489	MBBS, n=96	BAMS, n=135	BHMS, n=65	Paramedical, n=126	Nursing, n=67
Does a woman in a sexually active age group require a Pap smear?	Pre-lecture	104 (21%)	21 (22%)	17 (13%)	25 (38%)	26 (21%)	17 (25%)
	Post-lecture	386 (79%)	85 (89%)	91 (67%)	57 (45%)	98 (78%)	55 (82%)
What is the cost of HPV vaccine available	Pre-lecture	24 (5%)	1 (1%)	8 (6%)	7 (11%)	5 (8%)	3 (4%)
	Post-lecture	194 (40%)	20 (21%)	50 (37%)	46 (71%)	51 (78%)	27 (40%)

Following the educational lecture, 65% of MBBS students and 31% of nursing students agreed that sexually active women should opt for the HPV vaccine, with an overall agreement rate of 45%. Across all groups, a substantial majority (78% overall) acknowledged their vulnerability to cervical cancer without vaccination. Post-lecture, an impressive 91% of students expressed willingness to get vaccinated, and an even higher 97% were prepared to actively contribute to spreading awareness. These results highlight positive shifts in both understanding and proactive attitudes toward cervical cancer prevention (Table 6).

**Table 6:** Post-lecture practices toward HPV vaccine MBBS, Bachelor of Medicine, Bachelor of Surgery; BAMS, Bachelor of Ayurvedic Medicine and Surgery; BHMS, Bachelor of Homoeopathic Medicine and Surgery; HPV, human papillomavirus

Practices	Overall, n= 489	MBBS, n=96	BAMS, n=135	BHMS, n=65	Paramedical, n=126	Nursing, n=67
Should a sexually active female be offered HPV vaccine?	220 (45%)	62 (65%)	47 (55%)	35 (54%)	55 (44%)	21 (31%)
Are you at risk of infection?	383 (78%	77 (80%)	98 (73%)	52 (80%)	107 (85%)	49 (73%)
Want to get vaccinated post-lecture?	446 (91%)	87 (91%)	114 (84%)	61 (94%)	122 (97%)	62 (93%)
Will you spread awareness?	476 (97%)	95 (99%)	126 (93%)	65 (100%)	124 (98%)	66 (99%)

To increase awareness and acceptance of HPV vaccine in the community, various options were given to participants and they were asked to arrange according to their perceptions and preferences. MBBS, BHMS, paramedical, and nursing students chose inclusion of HPV vaccine in the NIS as the most critical measure to increase awareness and acceptance, while BAMS students opted for sex education as the most critical measure. Media campaign is a very important measure as per BAMS and paramedics (Table 7).

**Table 7:** Measures that can increase the awareness and acceptance of HPV vaccine in the general public according to the study participants A, most critical; B, very important; C, important; D, less important; E, least important; MBBS, Bachelor of Medicine, Bachelor of Surgery; BAMS, Bachelor of Ayurvedic Medicine and Surgery; BHMS, Bachelor of Homoeopathic Medicine and Surgery; HPV, human papillomavirus

Participants	Include in the National Immunization Schedule	Cost-cutting	Sex education	Timely advice by doctors, parents, and teachers	Media campaign
MBBS (96)	A	C	B	D	E
BAMS (135)	E	C	A	D	B
BHMS (65)	A	B	D	C	E
Paramedical (126)	A	C	D	E	B
Nursing (67)	A	C	D	B	E

When queried about the most critical barrier to the acceptance of the HPV vaccine, MBBS, BHMS, and paramedics students identified lack of awareness, while BAMS students underscored gender bias. Nursing students expressed the belief that cervical cancer is perceived as a disease of the poor as the primary barrier. A common secondary concern across all student groups was the high cost of the vaccine. This diversity in perceived barriers highlights the importance of tailored strategies to address specific concerns within each fraternity and underscores the multifaceted nature of improving HPV vaccine acceptance (Table 8).

**Table 8:** Barriers toward implementation and acceptability of HPV vaccine according to study participants A, most critical; B, very important; C, important; D, less important; E, least important; MBBS, Bachelor of Medicine, Bachelor of Surgery; BAMS, Bachelor of Ayurvedic Medicine and Surgery; BHMS, Bachelor of Homoeopathic Medicine and Surgery; HPV, human papillomavirus

Participants	High Cost	Lack of awareness	Sociocultural and religious barriers	Belief that HPV infection is more common in poor families only	Gender bias
MBBS	B	A	C	D	E
BAMS	B	C	D	E	A
BHMS	B	A	D	C	E
Paramedical	B	A	D	C	E
Nursing	B	C	D	A	E

## Discussion

Cervical cancer poses a significant health challenge in India. The current study, conducted among undergraduate medical students from diverse fields such as MBBS, BAMS, and BHMS within a private university, revealed a mean age of 21 years. A prior study on similar knowledge among students reported a slightly younger mean age of 19 years. Integrating knowledge and awareness programs with educational interventions holds promise for reducing HPV infection and effectively controlling cervical cancer in Indian women [16], while in a study conducted in south India, the mean age was 41 years [17]. The current study, conducted with predominantly unmarried participants (99%), contrasts with a similar study in Southern India where all participants were unmarried. In our study, 9% of participants had not heard of cervical cancer, and 28% were unaware of the vaccine's existence. In a comparable study, 9% of students had no knowledge of the vaccine. These findings highlight variations in awareness and knowledge levels among study participants [16].

In our current study, books and websites emerged as the preferred sources of information, presenting a departure from findings in various other studies. For instance, a Malaysian study highlighted word of mouth as the most preferred way, and television served as the primary source of information on the HPV vaccination program for respondents in certain studies. These disparities underscore the importance of tailoring information dissemination strategies to the unique preferences of diverse populations [18].

In a study conducted in Karnataka, overall awareness about cervical cancer and HPV was notably low, with only 15% of women reporting familiarity with cervical cancer and 36% having heard of HPV. Similarly, studies in both Karnataka and Himachal Pradesh indicated a moderate understanding of cervical cancer risk factors and symptoms among participants in pre-lecture questionnaires. These consistent findings underscore the widespread lack of awareness and knowledge regarding cervical cancer and HPV.

In the Himachal Pradesh study, a concerning discovery was made, with 76% of MBBS students responding that HPV infection has no symptoms. This underscores a critical gap in knowledge among healthcare students, emphasizing the urgent need for targeted educational interventions even within the medical community. Efforts to enhance awareness and understanding should be prioritized to address these knowledge gaps in diverse regions [19,20]. In the pre-lecture questionnaire, more than 50% of participants correctly identified that the HPV virus causes cervical cancer, aligning with findings from a Turkish study where 42% had heard about the HPV virus [21]. Approximately 54% recognized sexual transmission as the mode of transmission, consistent with another study where 50% identified sexual transmission [19]. In another study, 60% of participants responded correctly for transmission [16].

Notably, in an Indian multicentric study, the route of transmission was correctly answered by only 23% pre-lecture, but after the lecture, an impressive 99% responded accurately. These results underscore the impact of educational interventions in improving knowledge and awareness [15]. In almost all participant categories, correct responses regarding the ideal age for the HPV vaccine were notably low, totaling only 7% overall. Notably, in the Himachal Pradesh study, 63% of respondents opted for the "don't know" option, indicating a significant lack of accurate information about the recommended age for vaccination. These findings underscore a widespread gap in knowledge regarding the appropriate age for HPV vaccination, emphasizing the need for targeted educational efforts to address this specific aspect of awareness [20].

In the pre-lecture session of the present study, only 21% of students demonstrated awareness of a Pap smear. This contrasts with findings from a study conducted in Western India, where a mere 11% of participants were familiar with Pap smear. The disparity in awareness levels may be attributed to variations in the educational backgrounds of the two study participant groups. This highlights the influence of educational factors on knowledge levels and emphasizes the importance of tailored educational interventions to address specific awareness gaps [8]. In the current study, the most significant barrier identified was a lack of awareness. Interestingly, in a qualitative multicentric study across India, Peru, Uganda, and Vietnam, Indian participants ranked stories of adverse effects as the primary barrier and lack of awareness as the second most important barrier. This discrepancy could be attributed to the larger sample size and the qualitative nature of the latter study. This emphasizes the need for nuanced interpretations considering study design and participant demographics [22]. In the present study, the shift in correct responses from pre-lecture to post-lecture varied widely, ranging from 48% to 99% across different questions. Notably, 91% expressed willingness to get vaccinated post-lecture, and an impressive 99% were agreeable to actively spreading awareness about HPV.

In a multicentric Indian study using a pre- and post-educational lecture setup, participants initially demonstrated poor awareness about HPV infection and vaccination. However, post-health talks, there was a significant positive improvement, with nearly 100% correct responses in the knowledge domain. Importantly, 74.4% expressed agreement to get vaccinated, indicating a positive impact on both knowledge and vaccine acceptance [15].

Study limitations

The sample population may not reflect the knowledge and attitude of female students in the healthcare sector across all cities/states of India. The level of in-depth knowledge about HPV and HPV vaccine might differ among students in different educational categories in the healthcare sector. This is a self-designed questionnaire that may not be sufficient to address each and every aspect related to knowledge, attitude, and practice about HPV vaccine.

## Conclusions

The present study highlights a concerning gap in the knowledge among college students regarding cervical cancer. While they may be aware of its existence, their understanding of the disease remains limited. Moreover, HPV vaccination rates are alarmingly low among this demographic, leaving them vulnerable to the disease. Females working in the healthcare sector must be a role model for the young girls and their parents in the community. However, study findings offer a glimmer of hope. Following a brief educational lecture, we observed a significant increase in willingness among students to receive the HPV vaccine. This demonstrates the power of education in motivating individuals toward preventive measures.

## Figures and Tables

**Figure 2:**
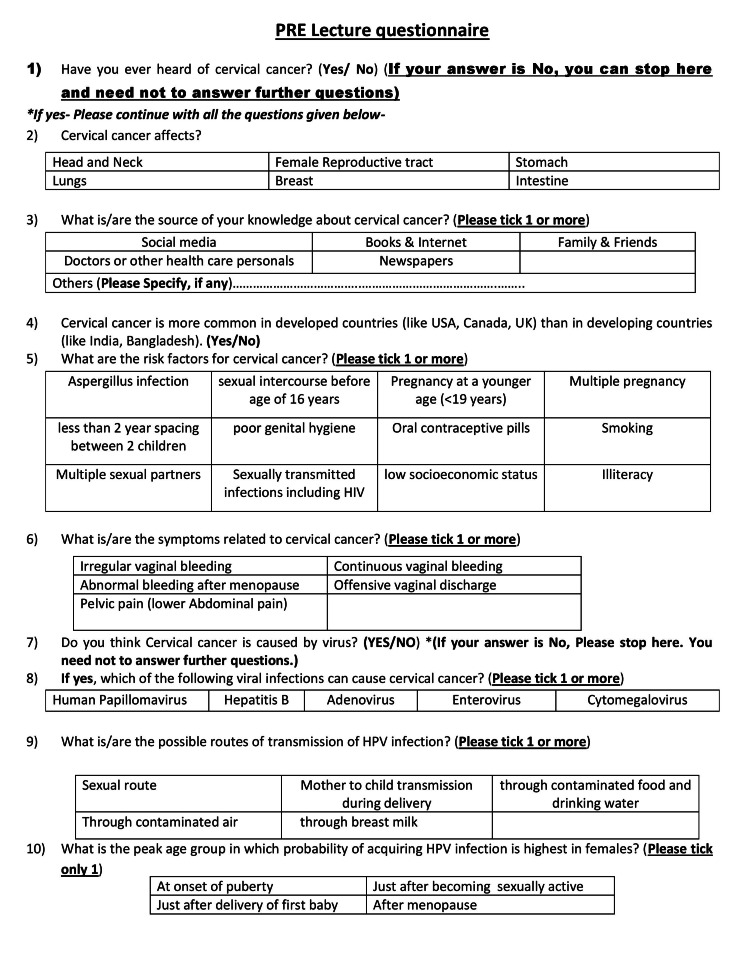
Pre-lecture questionnaire

**Figure 3:**
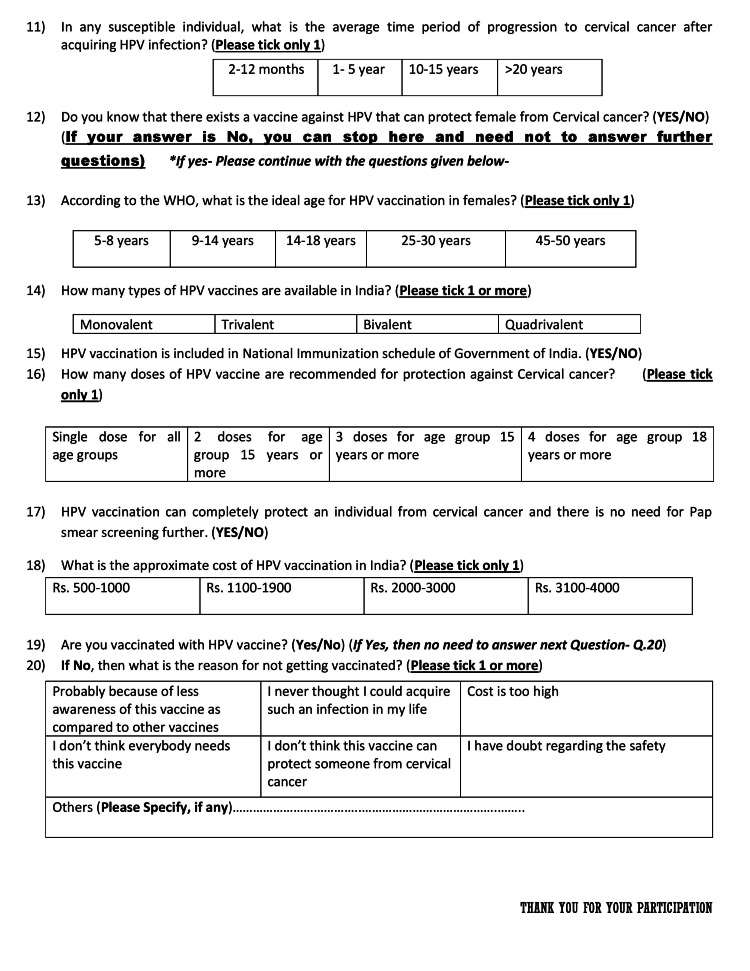
Pre-lecture questionnaire

**Figure 4:**
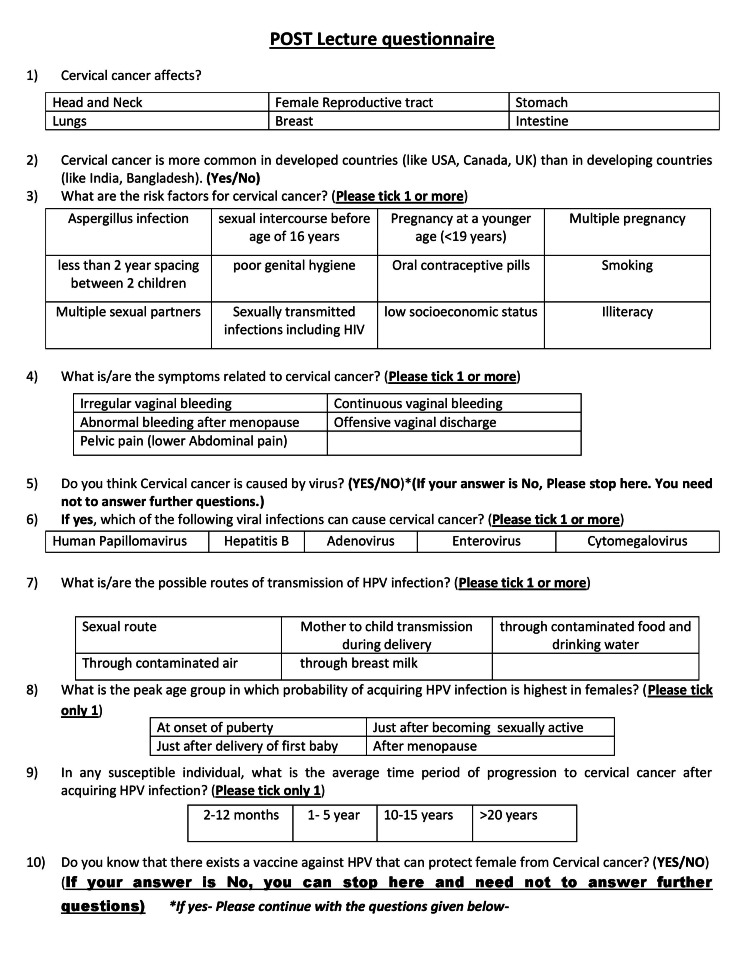
Post-lecture questionnaire

**Figure 5:**
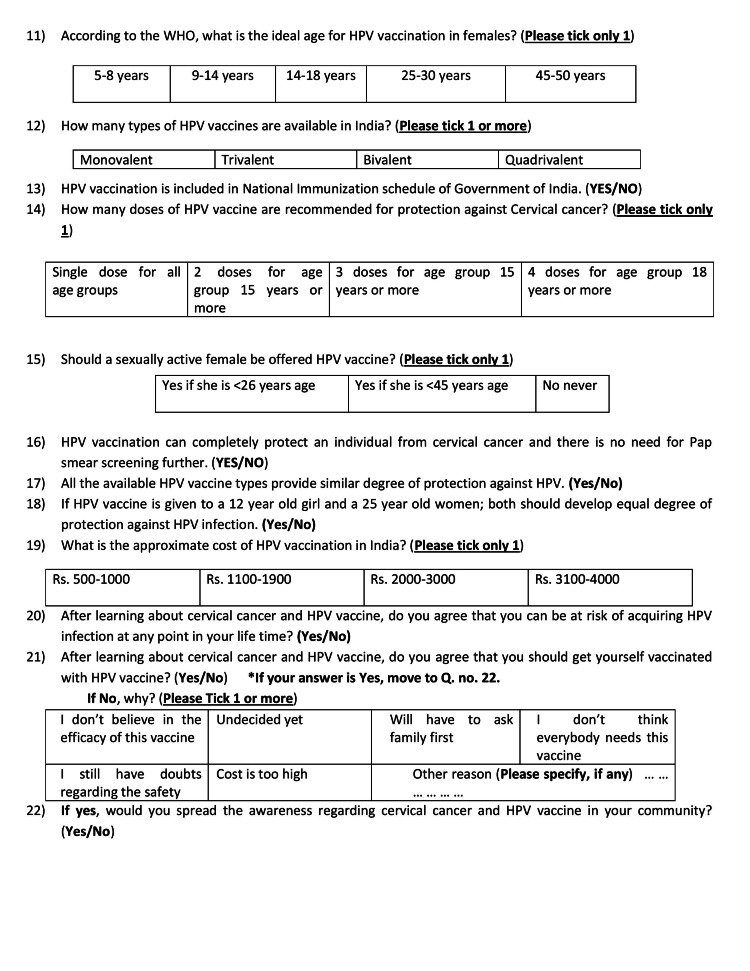
Post-lecture questionnaire

**Figure 6:**
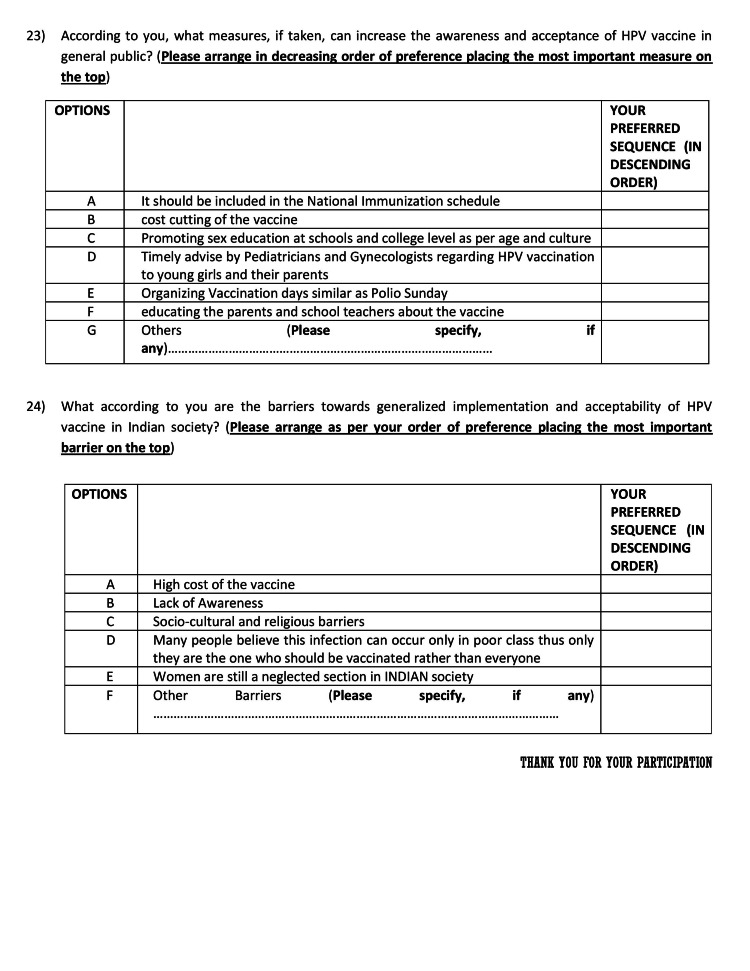
Post-lecture questionnaire

## Appendices

Appendix A: Pre-lecture questionnaire

Appendix B: Post-lecture questionnaire
